# Progress in Defining the Role of RSV in Allergy and Asthma: From Clinical Observations to Animal Models

**DOI:** 10.1080/10446670410001722131

**Published:** 2004-06

**Authors:** W. V. Kalina, L. J. Gershwin

**Affiliations:** 1 Department of Pathology Microbiology and Immunology School of Veterinary Medicine University of California Davis CA 95616 USA

## Abstract

Respiratory syncytial virus (RSV), an RNA virus in the family Paramyxoviridae, causes respiratory disease in humans. A closely related bovine RSV is responsible for a remarkably similar disease syndrome in young cattle. Severe RSV disease is characterized by bronchiolitis. The impact of RSV on human health is demonstrated annually when infants are admitted to the hospital in large numbers. Nearly every child will have been infected with RSV by the age of 3 years. While the disease is most severe in young infants and elderly people, it can re-infect adults causing mild upper respiratory tract disease throughout life. In addition, there is growing evidence that RSV infection may also predispose some children to the development of asthma. This is based on the observation that children who wheeze with RSV-induced bronchiolitis are more likely to develop into allergic asthmatics. Recent studies describe attempts to create an RSV induced asthma model in mice and other species; these have shown some degree of success. Such reports of case studies and animal models have suggested a wide range of factors possibly contributing to RSV induced asthma, these include timing of RSV infection with respect to allergen exposure, prior allergic sensitization, environmental conditions, exposure to endotoxin, and the genetic background of the person or animal. Herein, we primarily focus on the influence of RSV infection and inhalation of extraneous substances (such as allergens or endotoxin) on development of allergic asthma.


*Clinical & Developmental Immunology*, June 2004, Vol. 11 (2), pp. 113–119

Taylor & Francis healthsciences

### Progress in Defining the Role of RSV in Allergy and Asthma: From Clinical Observations to Animal Models

W.V. KALINA and L.J. GERSHWIN*

*Department of Pathology, Microbiology, and Immunology, School of Veterinary Medicine, University of California, Davis, CA 95616, USA*

Respiratory syncytial virus (RSV), an RNA virus in the family Paramyxoviridae, causes respiratory disease in humans. A closely related bovine RSV is responsible for a remarkably similar disease syndrome in young cattle. Severe RSV disease is characterized by bronchiolitis. The impact of RSV on human health is demonstrated annually when infants are admitted to the hospital in large numbers. Nearly every child will have been infected with RSV by the age of 3 years. While the disease is most severe in young infants and elderly people, it can re-infect adults causing mild upper respiratory tract disease throughout life. In addition, there is growing evidence that RSV infection may also predispose some children to the development of asthma. This is based on the observation that children who wheeze with RSV-induced bronchiolitis are more likely to develop into allergic asthmatics. Recent studies describe attempts to create an RSV induced asthma model in mice and other species; these have shown some degree of success. Such reports of case studies and animal models have suggested a wide range of factors possibly contributing to RSV induced asthma, these include timing of RSV infection with respect to allergen exposure, prior allergic sensitization, environmental conditions, exposure to endotoxin, and the genetic background of the person or animal. Herein, we primarily focus on the influence of RSV infection and inhalation of extraneous substances (such as allergens or endotoxin) on development of allergic asthma.

*Keywords*: RSV; Asthma TH2 atopic disease; IgE cells

### INTRODUCTION

Clinical observations that children who are infected with RSV and acquire bronchiolitis at an early age are more likely to develop childhood asthma are numerous (Sigurs *et al.*, 1995, 2000; Juntti *et al.*, 2003). Moreover, other reports have shown that allergens and environmental pollutant exposure can alter the outcome of RSV infection (Gurkan *et al.*, 2000; Makela *et al.*, 2003). In murine models, researchers have demonstrated increased airway hyperresponsiveness when allergen is administered before infection to achieve sensitization and then challenge exposure is performed during infection (Peebles *et al.*, 2001). This phenomenon has been demonstrated not only in the mouse but also in bovine models of RSV infection. A variety of different extraneous antigens have been employed to demonstrate this effect. These antigens include: cockroach allergen, ragweed, carbon black, *Micropolyspora faeni* (currently known as *Saccharopolyspora rectivirgula*), ovalbumin (OVA), and *Dermatophagoides farinae* (Df) (Leibovitz *et al.*, 1988; Gershwin and Giri, 1990, 1992, Gershwin *et al.*, 1994; Lukacs *et al.*, 2001; Peebles *et al.*, 2001; Foster *et al.*, 2003; Lambert *et al.*, 2003).

Unique aspects of RSV pathogenesis are important for understanding how RSV influences asthma pathogenesis, and how pathogenesis of RSV infection is influenced by inhalation of allergens. Thus, herein we discuss early and late immune responses to RSV and the interaction between inhaled allergens and RSV infection.

### RSV-cellular Attachment: A possible Instigator of Allergy

Unlike many other viruses, RSV's mechanism for entry into susceptible cells has not fully been elucidated. In a recent review, RSV was said to bind toll-like receptor 4 (TLR-4) prior to cell entry. TLR-4, CX3R1 (fractalkine receptor), sodium heparin and caveolin have been suggested as potential cellular receptors that are important for viral entry (Werling *et al.*, 1999; Karger *et al.*, 2001; Harris and Werling, 2003). TLR-4 binding to RSV does occur but is apparently not required to establish an infection. Kurt-Jones *et al.* demonstrated that RSV viral titers in the lungs of TLR-4 deficient C57BL10/ScCr mice

*Corresponding author. Tel.: +1-530-752-6643. Fax: +1-530-752-3349. E-mail: ljgershwin@ucdavis.edu

ISSN 1740-2522 print/ISSN 1740-2530 online © 2004 Taylor & Francis Ltd
DOI: 10.1080/10446670410001722131

were increased and IL-6 levels were suppressed. They also found that CD14 is necessary in conjunction with TLR-4 for stimulation of IL-6, TNFα, IL-8 and IL-1β production (Kurt-Jones *et al.*, 2000). The results of this study infer that the initial immune response to RSV may be instigated by TLR-4 binding to RSV.

TLR-4 expression is not normally seen in airway epithelial cells unless they are exposed to high amounts of LPS. Monick *et al.* evaluated the ability of A549 cells to produce IL-8 after exposure to LPS. They found that A549 cells produce very little IL-8 in response to LPS, which was due to low expression of TLR-4 (Monick *et al.*, 2003). They also showed that infection with live RSV induces A549 cells to increase both the expression of TLR-4 and the response to LPS. Under these conditions, RSV may prime airway epithelial cells to secrete pro-inflammatory mediators in response to RSV or bacteria. LPS has also been shown to cause mast cell secretion of IL-5, IL-10 and IL-13 but not TNFα via TLR-4 (Varadaradjalou *et al.*, 2003). Whether or not RSV induces mast cell activation by interaction with TLR-4 has not been elucidated. Nevertheless, RSV induced TLR-4 expression may hypersensitize epithelial cells to LPS and thereby instigate potent inflammatory responses. This is feasible because cross-linking of TLR-4, by RSV or LPS, also has an innate effect in increasing cellular expression of TLR-4. Increased TLR-4 expression on primary lung epithelial cells, caused by RSV, increases their ability to respond to lower amounts of bacterial or other LPS sources. This dual RSV and LPS exposure causes epithelial cells to secrete greater TNFα and/or IL-8 than either one alone (Monick *et al.*, 2003).

The contention that RSV primes airway epithelial cells to express TLR-4 has caused speculation that RSV infected epithelial cells are hypersensitive to LPS. This is of importance in cases of RSV induced asthma, where LPS administration almost always induces acute attacks in children (George *et al.*, 2001; Park *et al.*, 2001). It has been proposed that LPS contaminated grain dust, house dust and air pollution particles may act an adjuvants for responses to these allergens (Monick *et al.*, 2003). The LPS in these systems may instigate allergic sensitization, in airways, by mediating the production of IL-1 and enhance expression of co-stimulatory markers on antigen presenting cells. This mixture of cytokines and chemokines may attract lymphocytes to the lung and also, with increased expression of CD80/86 on monocytes, macrophages and epithelial cells, may enhance immune responses to allergens.

### Cell Types Involved in Early RSV Infection: Roles of Early Mediators in Th2 Responses

As mentioned above, monocytes, after interaction with RSV, express co-stimulatory molecules CD80/86 and present RSV peptides on MHCI and II (Soukup and Becker, 2003). Eosinophils also are active in RSV infections. They have been shown to present rhinoviral antigens to T cells and inactivate RSV by release of eosinophilic cationic protein (ECP) (Handzel *et al.*, 1998; Soukup and Becker, 2003). Exposure to the virus, promotes epithelial cells to release GM-CSF, G-CSF and RANTES, which attract more eosinophils and monocytes to the infection site. Furthermore, monocyte's release IL-16 when exposed to RSV, which attracts CD4+ T cells to the lung. Macrophages along with epithelial cells can secrete PGE2, IL-10 and IL-11 (Bartz *et al.*, 2002). These anti-inflammatory and Th1 blocking cytokines may always be produced in RSV infection as a negative feedback to the pro-inflammatory response. Allergen exposure, however, may enhance expression of the Th2 cytokines.

NK cells are the first lymphocyte sub-population to enter the lung during an RSV infection. By day 4, NK cells are the dominant lung lymphocyte population. They are responsible for the high amounts of IFNγ production seen early in RSV infections (Hussell and Openshaw, 1998).

IL-10, in addition to being immunosuppressive to Th1 responses, is produced early in RSV infections and has an anti-viral effect by increasing the expression of FasL on macrophages and cytotoxic T cells (Ruan *et al.*, 2001). Therefore, viral clearance is possible under Th2 conditions, however, immunological memory may be less protective. IL-10 is also necessary for sustaining mucosal tolerance by exerting its effects on alveolar macrophages and dendritic cells, however, in the presence of TLR-4 binding RSV, IL-10 may not offer enough suppression to prevent a TLR-4 mediated inflammatory response (Akbari *et al.*, 2001; Macaubas *et al.*, 2003). Secondary exposure to, or challenge with allergens under these conditions could likely lead to the rapid recruitment and proliferation of allergen specific Th2 memory cells whose cytokine mileu would indirectly affect responses to RSV.

### The Role of Mediators Induced during Late RSV Infection on Development of Th2 Responses to Allergens

IL-4, IL-5, IL-13 and IFNγ are usually produced in the lung several days after initiation of an RSV infection. Lung IL-10 and IFNγ gene expression plateau around day 3 and remain elevated until day 7 in a cotton rat model of RSV infection (Blanco *et al.*, 2002). IL-4 message, in lymph derived lymphocytes, has been detected from days 1 to 10, post infection in a bovine model of RSV infection; peripheral blood IL-13 appears on day 8 and remains elevated beyond day 12 in a BALB/c mouse RSV model (Gershwin *et al.*, 2000; Lukacs *et al.*, 2001). High levels of IL-5 have been noted, by ELISA, in bronchio-alveolar lavage fluid on day 7 in RSV infected BALB/c mice (Schwarze *et al.*, 1999a,b). These cytokines have been associated with enhanced or delayed clearance, depending on which cytokine is predominately expressed. In conjunction with allergen sensitization, IL-4, IL-5, IL-13 have been shown to be important in promoting airway hyperresponsiveness (Makela *et al.*, 2003; Park *et al.*, 2003). IL-10 is critical in mediating airway hyperresponsiveness in RSV infections without allergen. It has been shown that RSV infected IL-10 deficient mice produce less airway hyperresponsiveness, measured by lung resistance to increasing concentrations of methacholine, than wild type mice (Makela *et al.*, 2002). In general, RSV infection can induce expression of IL-4, IL-13, IL-5, IL-10, IL-12, IFNγ and others, but their relative proportions to one another can be influenced by allergens, pollution or dust in the environment (Gershwin *et al.*, 2000; Bartz *et al.*, 2002).

IL-4, IL-5, IL-10, IL-11 and IL-13 have been grouped in the Th2 class of cytokines that are most commonly associated with airway hyperresponsiveness in RSV infections. These cytokines contribute towards immune response modulation. IL-4 and IL-13, for instance, can promote B cell proliferation and IgE production, and IL-4 has been shown to inhibit nitric oxide (NO) production in A549 cells (Kao *et al.*, 2001). NO is important in regulating the IL-12/IL-10 balance secreted by alveolar macrophages. Overproduction of IL-4 can lead to large amounts of IL-10, which may help to promote IgE production. IL-10 induced anergy is commonly observed in cases where allergen is repeatedly administered, however, during viral infections, IL-4 and IFNγ increase expression of MHCII on alveolar macrophages. This increase in MHCII and antigen presentation may instigate immune responses to allergens in the lung (Jutel *et al.*, 2003; Macaubas *et al.*, 2003; Maurer *et al.*, 2003). IL-11 has also been implicated in airway hyperresponsiveness in RSV infections and may augment IL-4 and other Th2 responses to allergens (Einarsson *et al.*, 1996).

Th1 cytokines such as IFNγ, produced in RSV infection, participate in both viral clearance and pathology (Ostler *et al.*, 2002). In a study by Ostler *et al.* cytotoxic T cells, which require IFNγ for proliferation and activation, were thought to contribute to lung pathology. *In vivo* generated RSV M2 p85-90 specific CTL effectors that were adoptively transferred to RSV infected BALB/c mice promoted viral clearance with extensive peribronchiolar and intra-alveolar infiltrates (Ostler *et al.*, 2002). This pathological condition was observed clinically as reduced activity, loss of fur, hunch posture and weight loss. It has been proposed that CTLs do not immediately lyse virus infected cells. Instead, the target cell is signaled to secret pro-inflammatory mediators such as monocyte chemoattractant protein-1 (MCP-1) (Small *et al.*, 2001). Mucosal homing lymphocytes (CD54+ and CD102+) express IFNγ more often than IL-4 and may account for predominate Th1 responses reported in RSV infections (Tripp *et al.*, 2000). However, in a study by Spender *et al.*, BALB/c mice immunized with vaccinia virus expressing RSV G protein produced high amounts of BAL cell derived IL-5 mRNA, a Th2 cytokine, despite high IFNγ mRNA levels. The authors stated that increases in Th2 cytokines and lung eosinophilia can persist regardless of IFNγ producing Th1 cells (Spender *et al.*, 1998). Some researchers have proposed separate roles for two types of cytotoxic T cells that contribute to allergen induced airway hyperresponsiveness in RSV infections. One type is a CD8+ subset of short-lived anti-viral/IFNγ producing T cells and the other is a long-lived CD8+/IL-5 producing subset (Schwarze *et al.*, 1999a,b). The latter subset may recruit eosinophils to the lung long after resolution of an RSV infection. Whether or not both subsets of CTLs regulate each other's expression of cytokines is not know.

### Mechanisms of Allergen Induced RSV Immunomodulation

Researchers investigating the interaction of RSV with allergen are divided into two main groups: (1) those that are interested in how allergic sensitization and challenge can affect the outcome and severity (exacerbation) of RSV bronchiolitis and (2) those interested in the development of asthma subsequent to RSV infections. In the first case, investigators sensitize animals with allergen and challenge during RSV infection, and the second employs RSV infection followed by allergen aerosol. Both models induce airway hyperresponsiveness to varying degrees. Some have shown that RSV bronchiolitis is more severe in the first case. In several experimental systems, allergen sensitization preceded infection by several weeks and then challenge took place during RSV infection (Gershwin *et al.*, 1990; Gershwin and Giri, 1992; Barends *et al.*, 2002; Lambert *et al.*, 2003). These experimental systems employed allergens such as *Micropolyspora faeni* (currently known as *Saccharopolyspora rectivirgula*), Ovalbumin (OVA), or carbon black. To study RSV exacerbation, allergen sensitization must occur long before infection is to take place. This is needed to establish a Th2, IgE producing immune response to the allergen, which takes at least 2 weeks. After that time, IgE produced by lymph node or mucosal plasma cells will have sensitized tissue mast cells. When allergen challenge takes place the resulting activation of mast cells and release of mast cell byproducts (histamine, leukotrienes B4, C4, D4, E4, prostaglandins D2 and E2 and TNFα) cause a massive inflammatory tissue response and recruitment of granulocytes and lymphocytes. This reaction is characteristic of type I hypersensitivity. If this occurs during infection, the additive impact of chemokine production and resultant inflammation worsens disease. This has been documented in a bovine RSV model (Gershwin and Giri, 1992). RSV infection has also been shown to stimulate leukotriene release in nasal secretions of infants suffering from acute bronchiolitis (Volovitz *et al.*, 1988).

Th2 cytokines produced during allergen challenge, in conjunction with the previously mentioned pro-inflammatory mediators, can lead to dominating RSV Th2 responses. Allergen challenge during RSV infection has been shown to increase IL-5, IL-10, IL-13 and lung eosinophils. Although IL-10 expression is increased in

OVA sensitized/challenged and RSV infected mice, it does not seem to enhance airway hyperresponsiveness. This was demonstrated by Makela *et al.* using C57BL IL-10 knockout mice. They showed that there was no difference in airway hyperresponsiveness between IL-10 knockout mice and normal mice, and that elevated IL-5 and lung eosinophils, which were not induced by IL-10, were involved in airway hyperresponsiveness (Makela *et al.*, 2002). In the case of IL-13, the indirect manifestations of its release are increased expression of MCP-3, which triggers histamine release in mast cells, attraction of eosinophils and basophils, and increased monocyte derived chemokine (MDC) (Lukacs *et al.*, 2001). Release of these mediators can be initiated by PGD2, which is produced in allergic reactions. Honda *et al.* demonstrated that aerosolization with PGD2 1 day prior to allergen challenge enhances production of MDC, a chemoattractant in Th2 cells, as well as, IL-4 and IL-5 expression (Honda *et al.*, 2003). In addition, activated mast cells, as previously mentioned, secrete numerous Th2 cytokines. They concluded that PGD2 accelerates Th2 type inflammation by inducing MDC. Since RSV enhances PGD2 production, the combination of an allergic response and virus infection would be expected to enhance disease.

### Mechanisms of RSV Induced Allergen Immunomodulation

Allergen exposure prior to RSV infection worsens disease in animal models and is likely to play a role in human infections. However, children whom acquire a severe RSV infection at a very young age have a greater likelihood of developing asthma or allergic disease. In two cohort studies, children with severe RSV infections were more likely to produce IgE to common allergens after an infection. Sigurs *et al.* found that children, averaging $3\frac{1}{2}$ months old, whom develop severe RSV bronchiolitis have a greater likelihood of developing childhood asthma (Sigurs *et al.*, 1995). Children who developed RSV, early in life, were more likely to produce IgE to common food and inhalant allergens such as egg white, cat, birch and mite (Sigurs *et al.*, 1995). A cohort study done by Foster *et al.* also found that IgE, to common allergens, was more common after severe RSV infections in young children (Foster *et al.*, 1996). Furthermore, RSV infection after 2 years of age did not play a role in sensitization against aeroallergens (Foster *et al.*, 1996). This implies that RSV plays a pivotal role in influencing immune responses to allergens in young children.

Animal models have been used to investigate the role of RSV infection and subsequent allergic sensitization. In experiments, when RSV is administered prior to allergen aerosol, there is no immediate effect on clinical signs. RSV specific IgG and Th cell profile does not appear to be altered, however, some investigators have reported an increase in allergen specific IgG (Freihorst *et al.*, 1988; Gershwin *et al.*, 1994; Dakhama *et al.*, 1999). Dakhama *et al.* showed that guinea pigs infected with RSV and then aerosolized with OVA developed higher titers of OVA specific IgG1. Freihorst *et al.* demonstrated higher IgG in RSV infected OVA aerosolized BALB/c mice; Gershwin *et al.* reported a similar finding in BRSV infected calves subsequently aerosolized with *Micropolyspora faeni* (currently known as *Saccharopolyspora rectivirgula*). Whereas, others, using different OVA aerosol schedules (starting day 10 post infection) in BALB/c mice, have not noted an increase in allergen specific IgG but have observed increases in airway hyperresponsiveness (Schwarze *et al.*, 1997). O'Donnell *et al.* showed that aerosol of OVA during acute RSV infection (days 4–13) produced anaphylactic shock in all BALB/c mice when challenged with intradermal OVA 5 days after the last aerosol (O'Donnell and Openshaw, 1998). They also noted that the timing of aerosols influenced anaphylaxis and OVA specific IgG1 levels. Mice that were given OVA aerosols from days 11–20 and 18–27 post infection failed to undergo anaphylaxis on days 25 and 32, respectively when challenged intradermally with OVA. In addition, IgG1 levels were not affected. Intracellular production of IL-4 was enhanced by virus infection during exposure in this model.

IL-4 and IL-13 have been implicated in airway hyperresponsiveness (Schwarze *et al.*, 1997). Barends *et al.* noted that if allergen challenge takes place during an RSV infection, then IL-4 and IL-5 responses to the allergen become enhanced (Barends *et al.*, 2002). In addition, if IFNγR (Receptor) knockout mice are OVA sensitized and challenged during RSV infection, a strong IL-4/IL-13 response develops with increasing numbers of pulmonary eosinophils (Barends *et al.*, 2002). The levels of IL-4/IL-13 and pulmonary eosinophils are decreased significantly in OVA/RSV mice with functional IFNγR. Although airway hyperresponsiveness occurs in both models, the effect of allergen challenge is less in the mice that have functional IFNγR. In another study using mice, Lambert *et al.* showed that carbon black exposure prior to infection also results in higher Th2 cytokine IL-13 protein levels in BAL on days 4, 7 and 10. Decreases in IP-10, TNFα and IFNγ in carbon black/RSV infected mice were lower than RSV infected controls (Lambert *et al.*, 2003). These cytokines are important for the generation of Th1 and anti-viral responses and their suppression early in infection, as shown by Lambert *et al.*, may promote allergic responses in the lung.

In contrast, Peebles *et al.* found that mice immunized i.p. with OVA in alum and subsequently aerosolized with OVA on days −2 through 6 post RSV infection found no increase in lung IL-4, IL-5 or IL-13 mRNA expression despite greater airway hyperresponsiveness (Peebles *et al.*, 2001). There is an explanation for both results. Since two immune responses are taking place at once, and the RSV specific response has preceded the allergic response, the outcome of allergic disease may depend on the RSV immune phenotype. This could be influenced by any one or more of the following factors: prior allergen exposure, genetic predisposition to Th2 responses, different strains of mice in experimental models, prior history of microbial infections (hygiene hypothesis).

Whereas some investigators have argued that RSV infection immunomodulates allergenic responses to a Th1 phenotype, others have advocated Th2 or Th0 response augmentation. Leibovitz *et al.* aerosolized mice with ragweed allergen following RSV infection and measured higher amounts of both ragweed specific IgE and IgG in mice than non-infected controls (Leibovitz *et al.*, 1988). Noma *et al.* demonstrated that allergic responses are influenced by IL-2 produced in RSV infections, which can amplify allergen Th2 responses (Noma *et al.*, 1996). This, once again, points to the Th2 inducing potential of RSV, which is a point of conflict. Differences in data may be attributed to strains of mice that are used in the procedure. BALB/c mice tend to be more proficient in producing Th2 responses than other strains such as C57BL. There does seem to be agreement, for the most part, that RSV enhances allergic disease. Some differences in studies may be attributed to different mouse strains, but this does not explain situations where Th1 responses are observed to RSV in BALB/c mice. The question lies in how such a response instigates allergy. In response, some studies have suggested that RSV G protein specific CD4+T cells are strong producers of IL-3, IL-4 and IL-5; whereas, F and M protein specific T cells are more noted for IL-2 secretion (Alwan and Openshaw, 1993; Alwan *et al.*, 1994; Bembridge *et al.*, 1998). The majority of Th2 responses are directed to a secreted form of the G protein, which may be an evasive mechanism employed by RSV (Johnson *et al.*, 1998). F and M proteins are more immunogenic and may mask G protein specific responses when looking at RSV responses *in vitro* or *in vivo*. Nevertheless, G protein induced Th2 responses may be sufficient to evoke allergic responses in the host animal.

In marked contrast to those who believe RSV infection enhances allergic sensitization, some investigators have suggested that RSV infections very early in life are necessary for avoidance of atopic sensitization. Based on murine and human studies they have suggested that RSV instigates a strong Th1/IFNγ response and imply that its persistence during an early infection influences immune responses to allergens (Juntti *et al.*, 2003). Schwarze *et al.* demonstrated that BALB/c mice only infected with RSV displayed elevated IFNγ and decreased IL-4 and IL-5 on day 6 post infection in peribronchial lymph node mononuclear cells stimulated with either phorbol 12,13 dibutyrat/ionomycin or RSV (Schwarze *et al.*, 1997). High amounts of IFNγ and low IL-4 and IL-10 were also observed in RSV restimulated PBMCs in a human study of one hundred and eleven children under 6 months of age. This study compares the results of severe RSV vs. mild RSV infections and suggests other studies are biased because severe cases are compared with healthy non-infected children. Using this methodology, there was no statistical correlation between Th2 cytokines and disease severity (Brandenburg *et al.*, 2000). Other paramyxoviruses, such as measles virus have been scrutinized for their ability to decrease the prevalence of atopic disease and asthma. Industrialized countries where vaccines, antibiotics and anti-viral drugs are commonly used to prevent or treat infections reportedly are plagued by high incidences of asthma and atopy (Message and Johnston, 2002). In addition, rhinovirus infections, which are most prevalent in infants under 6 months old, have been suggested, along with RSV, to promote early Th1 responses (van Benten *et al.*, 2003).

### CONCLUSION

The influence of RSV infection on allergic sensitization and the influence of allergic sensitization on RSV bronchiolitis have been examined using relevant studies. While the effects of LPS and allergen exposure on pathogenesis of RSV infection have been well studied in mouse models, the cellular and molecular events that determine the ultimate outcome of the immune response remain to be fully elucidated. Indeed, mechanisms that favor the role of RSV in enhancing allergic sensitization are very likely not the same as those that facilitate asthma exacerbation when the allergic asthmatic child is infected with RSV. Further studies in other appropriate model systems such as the rhesus monkey/RSV and bovine calf/BRSV species may help to elucidate the complex interaction between virus, host and allergen.

### *References*

Akbari, O., DeKruyff, R.H. and Umetsu, D.T. (2001) “Pulmonary dendritic cells producing IL-10 mediate tolerance induced by respiratory exposure to antigen”, *Nat. Immunol.* **2**(8), 725–731.

Alwan, W.H. and Openshaw, P.J. (1993) “Distinct patterns of T- and B-cell immunity to respiratory syncytial virus induced by individual viral proteins”, *Vaccine* **11**(4), 431–437.

Alwan, W.H., Kozlowska, W.J. and Openshaw, P.J. (1994) “Distinct types of lung disease caused by functional subsets of antiviral T cells”, *J. Exp. Med.* **179**(1), 81–89.

Barends, M., Boelen, A., de Rond, L., Kwakkel, J., Bestebroer, T., Dormans, J., Neijens, H. and Kimman, T. (2002) “Influence of respiratory syncytial virus infection on cytokine and inflammatory responses in allergic mice”, *Clin. Exp. Allergy* **32**(3), 463–471.

Bartz, H., Buning-Pfaue, F., Turkel, O. and Schauer, U. (2002) “Respiratory syncytial virus induces prostaglandin E2, IL-10 and IL-11 generation in antigen presenting cells”, *Clin. Exp. Immunol.* **129**(3), 438–445.

Bembridge, G.P., Garcia-Beato, R., Lopez, J.A., Melero, J.A. and Taylor, G. (1998) “Subcellular site of expression and route of vaccination influence pulmonary eosinophilia following respiratory syncytial virus challenge in BALB/c mice sensitized to the attachment G protein”, *J. Immunol.* **161**(5), 2473–2480.

van Benten, I., Koopman, K., Niesters, B., Hop, W., van Middelkoop, B., de Waal, L., *et al.* (2003) “Predominance of rhinovirus in the nose of symptomatic and asymptomatic infants”, *Pediatr. Allergy Immunol.* **14**, 363–370.

Blanco, J.C., Richardson, J.Y., Darnell, M.E., Rowzee, A., Pletneva, L., Porter, D.D. and Prince, G.A. (2002) “Cytokine and chemokine gene expression after primary and secondary respiratory syncytial virus infection in cotton rats”, *J. Infect. Dis.* **185**(12), 1780–1785.

Brandenburg, A.H., Kleinjan, A., van het Land, B., Moll, H.A., *et al.* (2000) “Type 1-like immune response is found in children with respiratory synytial virus infection regardless of clinical severity”, *J. Med. Virol.* **62**, 267–277.

Dakhama, A., Bramley, A.M., Chan, N.G., McKay, K.O., Schellenberg, R.R. and Hegele, R.G. (1999) "Effect of respiratory syncytial virus on subsequent allergic sensitization to ovalbumin in guinea-pigs", *Eur. Respir. J.* **13**(5), 976–982.

Einarsson, O., Geba, G.P., Zhu, Z., Landry, M. and Elias, J.A. (1996) "Interleukin-11: stimulation *in vivo* and *in vitro* by respiratory viruses and induction of airways hyperresponsiveness", *J. Clin. Investig.* **97**(4), 915–924.

Foster, J., Tacke, U., Drebs, H., Streckert, H.J., Werchau, H., Bergmann, R.L., Schulz, J., Lau, S. and Wahn, U. (1996) "Respiratory syncytial virus infection: its role in aeroallergen sensitization during the first two years of life", *Pediatr. Allergy Immunol.* **7**(2), 55–60.

Foster, S., Bedford, K.J., Gould, M.E., Coward, W.R. and Hewitt, C.R. (2003) "Respiratory syncytial virus infection and virus-induced inflammation are modified by contaminants of indoor air", *Immunology* **108**(1), 109–115.

Freihorst, J., Piedra, P.A., Okamoto, Y. and Ogra, P.L. (1988) "Effect of respiratory syncytial virus infection on the uptake of and immune response to other inhaled antigens", *Proc. Soc. Exp. Biol. Med.* **188**(2), 191–197.

George, C.L., Jin, H., Wohlford-Lenane, C.L., O'Neill, M.E., Phipps, J.C., O'Shaughnessy, P., Kline, J.N., Thorne, P.S. and Schwartz, D.A. (2001) "Endotoxin responsiveness and subchronic grain dust-induced airway disease", *Am. J. Physiol. Lung Cell Mol. Physiol.* **280**(2), L203–L213.

Gershwin, L.J. and Giri, S.N. (1992) "Effects of allergen challenge on plasma concentrations of prostaglandins, thromboxane B2, and histamine in calves infected with bovine respiratory syncytial virus", *Am. J. Vet. Res.* **53**(9), 1670–1674.

Gershwin, L.J., Dungworth, D.L., Himes, S.R. and Friebertshauser, K.E. (1990) "Immunoglobulin E responses and lung pathology resulting from aerosol exposure of calves to respiratory syncytial virus and micropolyspora faeni", *Int. Arch. Allergy Appl. Immunol.* **92**, 293–300.

Gershwin, L.J., Himes, S.R., Dungworth, D.L., Giri, S.N., Friebertshauser, K.E. and Camacho, M. (1994) "Effect of bovine respiratory syncytial virus infection on hypersensitivity to inhaled micropolyspora faeni", *Int. Arch. Allergy Immunol.* **104**(1), 79–91.

Gershwin, L.J., Gunther, R.A., Anderson, M.L., Woolums, A.R., McArthur-Vaughan, K., Randel, E., Boyle, G.A., Friebertshauser, K.E. and McInturff, P.S. (2000) "Bovine respiratory syncytial virus-specific IgE is associated with interleukin-2 and -4 and interferon-$\gamma$ expression in pulmonary lymp of experimentally infected calves", *AJVR* **61**(3), 291–298.

Gurkan, F., Kiral, A., Dagli, E. and Karakoc, F. (2000) "The effect of passive smoking on the development of respiratory syncytial virus bronchiolitis", *Eur. J. Epidemiol.* **16**(5), 465–468.

Handzel, Z.T., Busse, W.W., Sedgwick, J.B., Vrtis, R., Lee, W.M., Kelly, E.A. and Gern, J.E. (1998) "Eosinophils bind rhinovirus and activate virus-specific T cells", *J. Immunol.* **160**(3), 1279–1284.

Harris, J. and Werling, D. (2003) "Binding and entry of respiratory syncytial virus into host cells and initiation of the innate immune response", *Cell Microbiol.* **5**(10), 671–680.

Honda, K., Arima, M., Cheng, G., Taki, S., Hirata, H., Eda, F., Fukushima, F., Yamaguchi, B., Hatano, M., Tokuhisa, T. and Fukuda, T. (2003) "Prostaglandin D2 reinforces Th2 type inflammatory responses of airways to low-dose antigen through bronchial expression of macrophage-derived chemokine", *J. Exp. Med.* **198**(4), 533–543.

Hussell, T. and Openshaw, P.J. (1998) "Intracellular IFN-gamma expression in natural killer cells precedes lung CD8+ T cell recruitment during respiratory syncytial virus infection", *J. Gen. Virol.* **79**(Pt 11), 2593–2601.

Johnson, T.R., Johnson, J.E., Roberts, S.R., Wertz, G.W., Parker, R.A. and Graham, B.S. (1998) "Priming with secreted glycoprotein G of respiratory syncytial virus (RSV) augments interleukin-5 production and tissue eosinophilia after RSV challenge", *J. Virol.* **72**(4), 2871–2880.

Juntti, H., Kokkonen, J., Dunder, T., Renko, M., Niinimaki, A. and Uhari, M. (2003) "Association of an early respiratory syncytial virus infection and atopic allergy", *Allergy* **58**(9), 878–884.

Jutel, M., Akdis, M., Budak, F., Aebischer-Casaulta, C., Wrzyszcz, M., Blaser, K. and Akdis, C.A. (2003) "IL-10 and TGF-beta cooperate in the regulatory T cell response to mucosal allergens in normal immunity and specific immunotherapy", *Eur. J. Immunol.* **33**(5), 1205–1214.

Kao, Y.J., Piedra, P.A., Larsen, G.L. and Colasurdo, G.N. (2001) "Induction and regulation of nitric oxide synthase in airway epithelial cells by respiratory syncytial virus", *Am. J. Respir. Crit. Care Med.* **163**(2), 532–539.

Karger, A., Schmidt, U. and Buchholz, U.J. (2001) "Recombinant bovine respiratory syncytial virus with deletions of the G or SH genes: G and F proteins bind heparin", *J. Gen. Virol.* **82**(Pt 3), 631–640.

Kurt-Jones, E.A., Popova, L., Kwinn, L., Haynes, L.M., Jones, L.P., Tripp, R.A., Walsh, E.E., Freeman, M.W., Golenbock, D.T., Anderson, L.J. and Finberg, R.W. (2000) "Pattern recognition receptors TLR4 and CD14 mediate response to respiratory syncytial virus", *Nat. Immunol.* **1**(5), 398–401.

Lambert, A.L., Mangum, J.B., DeLorme, M.P. and Everitt, J.I. (2003) "Ultrafine carbon black particles enhance respiratory syncytial virus-induced airway reactivity, pulmonary inflammation, and chemokine expression", *Toxicol. Sci.* **72**(2), 339–346.

Leibovitz, E., Freihorst, J., Piedra, P.A. and Ogra, P.L. (1988) "Modulation of systemic and mucosal immune responses to inhaled ragweed antigen in experimentally induced infection with respiratory syncytial virus implication in virally induced allergy", *Int. Arch. Allergy Appl. Immunol.* **86**(1), 112–116.

Lukacs, N.W., Tekkanat, K.K., Berlin, A., Hogaboam, C.M., Miller, A., Evanoff, H., Lincoln, P. and Maassab, H. (2001) "Respiratory syncytial virus predisposes mice to augmented allergic airway responses via IL-13-mediated mechanisms", *J. Immunol.* **167**(2), 1060–1065.

Macaubas, C., DeKruyff, R.H. and Umetsu, D.T. (2003) "Respiratory tolerance in the protection against asthma", *Curr. Drug Targets Inflamm. Allergy* **2**(2), 175–186.

Makela, M.J., Kanehiro, A., Dakhama, A., Borish, L., Joetham, A., Tripp, R., Anderson, L. and Gelfand, E.W. (2002) "The failure of interleukin-10-deficient mice to develop airway hyperresponsiveness is overcome by respiratory syncytial virus infection in allergen-sensitized/challenged mice", *Am. J. Respir. Crit. Care Med.* **165**(6), 824–831.

Makela, M.J., Tripp, R., Dakhama, A., Park, J.W., Ikemura, T., Joetham, A., Waris, M., Anderson, L.J. and Gelfand, E.W. (2003) "Prior airway exposure to allergen increases virus-induced airway hyperresponsiveness", *J. Allergy Clin. Immunol.* **112**(5), 861–869.

Maurer, M., Seidel-Guyenot, W., Metz, M., Knop, J. and Steinbrink, K. (2003) "Critical role of IL-10 in the induction of low zone tolerance to contact allergens", *J. Clin. Investig.* **112**(3), 432–439.

Message, S.D. and Johnston, S.L. (2002) "Viruses in asthma", *Br. Med. Bull.* **61**, 29–43.

Monick, M.M., Yarovinsky, T.O., Powers, L.S., Butler, N.S., Carter, A.B., Gudmundsson, G. and Hunninghake, G.W. (2003) "Respiratory syncytial virus up-regulates TLR4 and sensitizes airway epithelial cells to endotoxin", *J. Biol. Chem.* **278**(52), 53035–53044.

Noma, T., Mori, A. and Yoshizawa, I. (1996) "Induction of allergen-specific IL-2 responsiveness of lymphocytes after respiratory syncytial virus infection and prediction of onset of recurrent wheezing and bronchial asthma", *J. Allergy Clin. Immunol.* **98**(4), 816–826.

O'Donnell, D.R. and Openshaw, P.J. (1998) "Anaphylactic sensitization to aeroantigen during respiratory virus infection", *Clin. Exp. Allergy* **28**(12), 1501–1508.

Ostler, T., Davidson, W. and Ehl, S. (2002) "Virus clearance and immunopathology by CD8+ T cells during infection with respiratory syncytial virus are mediated by IFN$\gamma$", *Eur. J. Immunol.* **32**, 2117–2123.

Park, J.H., Gold, D.R., Spiegelman, D.L., Burge, H.A. and Milton, D.K. (2001) "House dust endotoxin and wheeze in the first year of life", *Am. J. Respir. Crit. Care Med.* **163**(2), 322–328.

Park, J.W., Taube, C., Yang, E.S., Joetham, A., Balhorn, A., Takeda, K., Miyahara, N., Dakhama, A., Donaldson, D.D. and Gelfand, E.W. (2003) "Respiratory syncytial virus-induced airway hyperresponsiveness is independent of IL-13 compared with that induced by allergen", *J. Allergy Clin. Immunol.* **112**(6), 1078–1087.

Peebles, R.S., Jr., Sheller, J.R., Collins, R.D., Jarzecka, A.K., Mitchell, D.B., Parker, R.A. and Graham, B.S. (2001) "Respiratory syncytial virus infection does not increase allergen-induced type 2 cytokine production, yet increases airway hyperresponsiveness in mice", *J. Med. Virol.* **63**(2), 178–188.

Ruan, Y., Okamoto, Y., Matsuzaki, Z., Endo, S., Matsuoka, T., Kohno, T., Chazono, H., Eiko, I., Tsubota, K. and Saito, I. (2001) "Suppressive effect of locally produced interleukin-10 on respiratory syncytial virus infection", *Immunology* **104**(3), 355–360.

Schwarze, J., Hamelmann, E., Bradley, K.L., Takeda, K. and Gelfand, E.W. (1997) "Respiratory syncytial virus infection results in airway hyperresponsiveness and enhanced airway sensitization to allergen", *J. Clin. Investig.* **100**(1), 226–233.

Schwarze, J., Makela, M., Cieslewicz, G., Dakhama, A., Lahn, M., Ikemura, T., Joetham, A. and Gelfand, E.W. (1999a) "Transfer of the enhancing effect of respiratory syncytial virus infection on subsequent allergic airway sensitization by T lymphocytes", *J. Immunol.* **163**(10), 5729–5734.

Schwarze, J., Cieslewicz, G., Joetham, A., Ikemura, T., Hamelmann, E. and Gelfand, E.W. (1999b) "CD8 T cells are essential in the development of respiratory syncytial virus-induced lung eosinophilia and airway hyperresponsiveness", *J. Immunol.* **162**(7), 4207–4211.

Sigurs, N., Bjarnason, R., Sigurbergsson, F., Kjellman, B. and Bjorksten, B. (1995) "Asthma and immunoglobulin E antibodies after respiratory syncytial virus bronchiolitis: a prospective cohort study with matched controls", *Pediatrics* **95**(4), 500–505.

Sigurs, N., Bjarnason, R., Sigurbergsson, F. and Kjellman, B. (2000) "Respiratory syncytial virus bronchiolitis in infancy is an important risk factor for asthma and allergy at age 7", *Am. J. Respir. Crit. Care Med.* **161**(5), 1501–1507.

Small, B.A., Dressel, S.A., Lawrence, C.W., Drake, D.R., Stoler, M.H., III, Enelow, M.H. and Braciale, T.J. (2001) "CD8(+) T cell-mediated injury *in vivo* progresses in the absence of effector T cells", *J. Exp. Med.* **194**(12), 1835–1846.

Soukup, J.M. and Becker, S. (2003) "Role of monocytes and eosinophils in human respiratory syncytial virus infection *in vitro*", *Clin. Immunol.* **107**(3), 178–185.

Spender, L.C., Hussell, T. and Openshaw, P.J. (1998) "Abundant IFN-gamma production by local T cells in respiratory syncytial virus-induced eosinophilic lung disease", *J. Gen. Virol.* **79**(Pt 7), 1751–1758.

Tripp, R.A., Moore, D. and Anderson, L.J. (2000) "TH(1)- and TH(2)-TYPE cytokine expression by activated t lymphocytes from the lung and spleen during the inflammatory response to respiratory syncytial virus", *Cytokine* **12**(6), 801–807.

Varadaradjalou, S., Feger, F., Thieblemont, N., Hamouda, N.B., Pleau, J.M., Dy, M. and Arock, M. (2003) "Toll-like receptor 2 (TLR2) and TLR4 differentially activate human mast cells", *Eur. J. Immunol.* **33**(4), 899–906.

Volovitz, B., Welliver, R.C., De Castro, G., Krystofik, D.A. and Ogra, P.L. (1988) "The release of leukotrienes in the respiratory tract during infection with respiratory syncytial virus: role in obstructive airway disease", *Pediatr. Res.* **24**(4), 504–507.

Werling, D., Hope, J.C., Chaplin, P., Collins, R.A., Taylor, G. and Howard, C.J. (1999) "Involvement of caveolae in the uptake of respiratory syncytial virus antigen by dendritic cells", *J. Leukoc. Biol.* **66**(1), 50–58.

